# Inhibitory role of TRIP-Br1 oncoprotein in anticancer drug-mediated programmed cell death via mitophagy activation

**DOI:** 10.7150/ijbs.72138

**Published:** 2022-05-29

**Authors:** Samil Jung, Davaajargal Myagmarjav, Taeyeon Jo, Soonduk Lee, Songyi Han, Nguyen Thi Ngoc Quynh, Nguyen Hai Anh, Son Hai Vu, Yeongseon Choi, Myeong-Sok Lee

**Affiliations:** Division of Biological Sciences, Sookmyung Women's University, Seoul, 14310, South Korea

**Keywords:** Cancer, Mitochondria, Autophagy, Mitophagy, TRIP-Br1

## Abstract

Chemotherapy has been widely used as a clinical treatment for cancer over the years. However, its effectiveness is limited because of resistance of cancer cells to programmed cell death (PCD) after treatment with anticancer drugs. To elucidate the resistance mechanism, we initially focused on cancer cell-specific mitophagy, an autophagic degradation of damaged mitochondria. This is because mitophagy has been reported to provide cancer cells with high resistance to anticancer drugs. Our data showed that TRIP-Br1 oncoprotein level was greatly increased in the mitochondria of breast cancer cells after treatment with various anticancer drugs including staurosporine (STS), the main focus of this study. STS treatment increased cellular ROS generation in cancer cells, which triggered mitochondrial translocation of TRIP-Br1 from the cytosol via dephosphorylation of TRIP-Br1 by protein phosphatase 2A (PP2A). Up-regulated mitochondrial TRIP-Br1 suppressed cellular ROS levels. In addition, TRIP-Br1 rapidly removed STS-mediated damaged mitochondria by activating mitophagy. It eventually suppressed STS-mediated PCD via degradation of VDACI, TOMM20, and TIMM23 mitochondrial membrane proteins. TRIP-Br1 enhanced mitophagy by increasing expression levels of two crucial lysosomal proteases, cathepsins B and D. In conclusion, TRIP-Br1 can suppress the sensitivity of breast cancer cells to anticancer drugs by activating autophagy/mitophagy, eventually promoting cancer cell survival.

## Introduction

Most anticancer drugs currently available target apoptosis. However, many malignant cancer cells have acquired resistance to apoptosis due to defective or absent apoptosis-related genes or signaling pathways, such as p53 mutations in many different types of cancer cells and the lack of caspase 3 in MCF7 breast cancer cell line. Determining novel targets is essential to increase the sensitivity of cancer cells to anticancer drugs. Targeting various types of PCD concurrently in addition to apoptosis is one of strategies to increase cancer cell sensitivity to anticancer drugs. Eukaryotic cells undergo more than 11 different types of PCD, including apoptosis, necrosis/necroptosis, and anoikis depending on the stimuli and environments 1,2. All known types of PCD are interconnected via multiple signaling pathways, in which mitochondria play a vital regulatory role 3. Thus, understanding the precise mechanism in mitochondria-mediated PCD would be important for controlling the resistance of cancer cells to anticancer drugs.

Toxicities of most chemotherapeutic agents are attributed to the induction of mitochondrial dysfunction 4. Mitochondrial-mediated PCD induced by anticancer treatments is often associated with decreases of ATP production and mitochondrial membrane potential (MMP), and with an increase in reactive oxygen species (ROS) generation 5-14. These changes can increase mitochondrial malfunction, triggering the release of pro-apoptotic proteins (such as cytochrome c) or pro-necroptotic proteins (such as CypA) from damaged mitochondria, eventually resulting in PCD. Therefore, rapid clearance of damaged mitochondria eventually inhibits PCD. Especially, intracellular ROS are major factors that decide cell fate 15,16. ROS at low concentrations are required for cell survival by acting as signaling molecules. Higher ROS levels can cause mild oxidative stress, which removes damaged mitochondria for cell survival 17. Further increase in ROS level can lead to apoptosis and sudden necrosis/necroptosis 9,18-21.

Dysfunctional or damaged mitochondria can be removed mainly by mitophagy or mitoptosis (mitochondrial suicide) 22. Mitophagy has been suggested to be highly activated in cancer cells in response to various stressful conditions 23. Mitophagy is mediated by autophagy-related proteins and lysosomal components. Damaged mitochondria are engulfed by autophagosomes and eventually degraded by lysosomes. Many studies have proposed that mitophagy has a dual role: 1) mitophagy prevents tumorigenesis of normal cells by removing abnormal mitochondria and therefore protecting cells from disordered mitochondrial metabolism; and 2) mitophagy promotes survival of cancer cells by suppressing sensitivity to chemotherapy 22,24-26. Therefore, targeting cancer-specific mitophagy might be a promising strategy to design effective chemotherapies. In fact, manipulating autophagy/mitophagy is currently considered as an attractive therapeutic strategy in cancer. However, its role in cancer remains unclear and strategies in cancer therapy remain confusing.

To elucidate the mechanism of cancer-specific mitophagy, we initially focused on TRIP-Br1 (also known as SERTAD1/SEI-1/SEI1/p34^SEI-1^) protein. TRIP-Br1 is a member of TRIP-Br family proteins that share the N-terminal SERTA domain. Our preliminary data have revealed that TRIP-Br1 is localized to the nucleus, cytoplasm, and mitochondria 27, where it exerts different cellular functions as a multifunctional protein. TRIP-Br1 is involved in transcriptional regulation, cell cycle, cell differentiation, senescence, and PCD regulation 27-39. Overexpressed in many different types of cancer cells and cancer patients, TRIP-Br1 plays a critical role in tumorigenesis as an oncogene 30. Our previous data have shown that TRIP-Br1 expression is greatly increased under different types of PCD-inducing stressful conditions, such as anticancer treatment, nutrient starvation, and hypoxia 27,40. Up-regulated TRIP-Br1 suppresses three types of PCD, including apoptosis, necrosis/necroptosis, and anoikis 27,28,40. For example, TRIP-Br1 inhibits apoptosis of breast cancer cells upon treatment with anticancer drugs (e.g., staurosporine, etoposide, and cisplatin) by stabilizing XIAP E3 ligase 28. TRIP-Br1/XIAP also represses necroptosis under nutrient/serum starvation 40. Our unpublished data showed that TRIP-Br1 also could suppress anoikis under three-dimentioanl suspended culture. Nevertheless, the exact mechanism involved in the effect of TRIP-Br1 on mitochondrial-mediated PCD remains unknown.

The current study shows that TRIP-Br1 oncogenic protein contributes to high cellular resistance to anticancer drug treatment via activation of mitophagy.

## Results

### Mitochondrial distribution/sub-localization of TRIP-Br1 protein in normal breast and cancer cell lines

**Figure 1:**
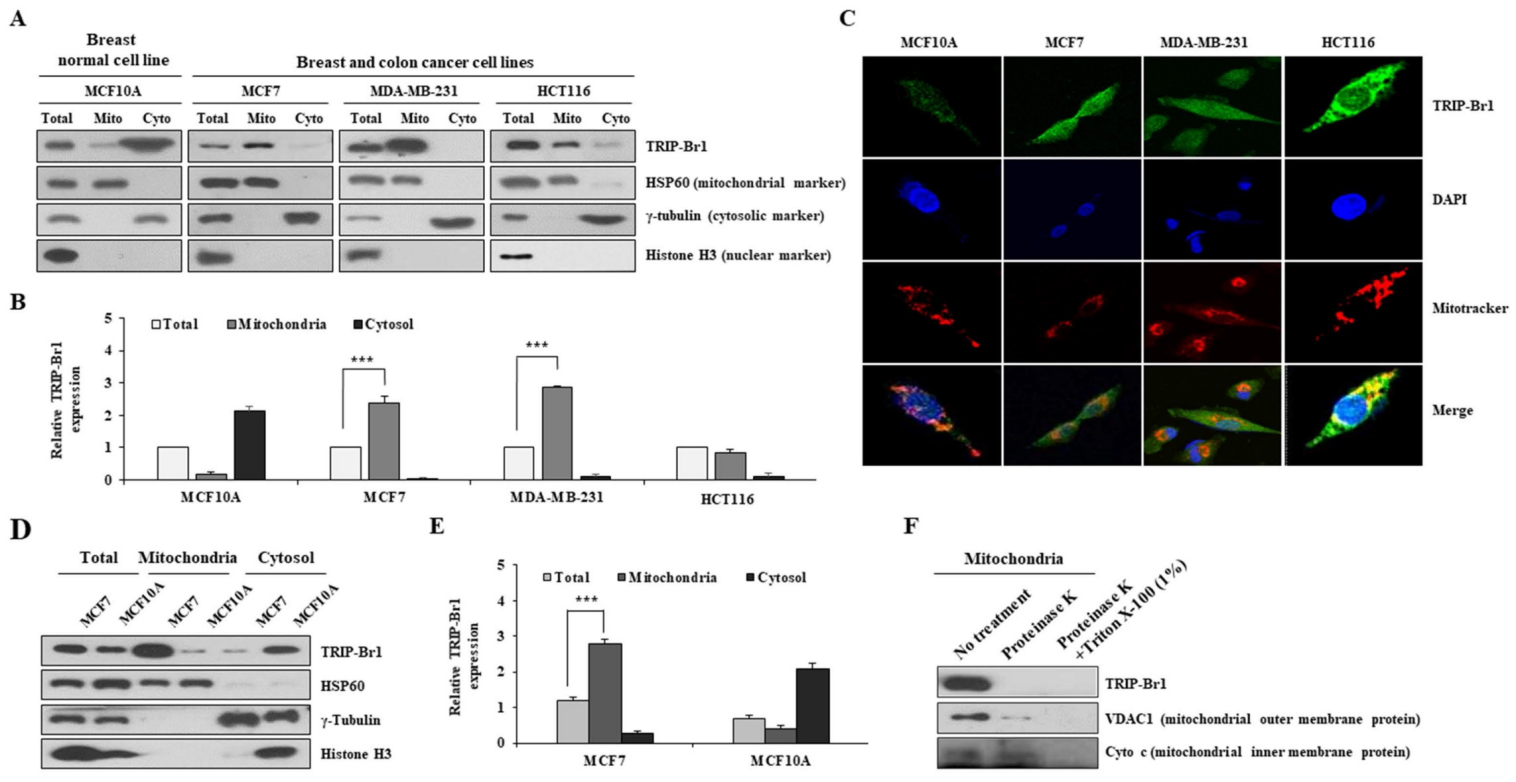
** Subcellular distribution of TRIP-Br1 protein in normal breast or cancer cell line and mitochondrial localization of TRIP-Br1. A-B** Indicated cell lines were used in mitochondrial fractionation as described in Materials and Methods. TRIP-Br1 protein level in each fraction was quantified via immunoblotting analyses. The following proteins were used as cellular markers: HSP60 as a mitochondrial marker, γ-tubulin as a cytosolic marker, and histone H3 as a nuclear marker. Amount of protein loaded: 15 µg for total protein, 2.5 µg for mitochondrial (Mito), and 15 µg for cytosolic (Cyto) fraction. Data are expressed as mean ± SD (n = 3; ***, *p* < 0.001). **C** Indicated four cell lines were stained with TRIP-Br1 (green) for 24 h, incubated with 1 µM of Mitotracker (red) for 30 min, and fixed with 4% paraformaldehyde. DAPI (blue) was used as a nuclear marker. Resulting cells were visualized with a confocal microscope. **D-E** MCF7 and MCF10A cell lines were cultured for 24 h and then used for mitochondrial fractionation. TRIP-Br1 protein level in each fraction was determined by western blot analysis. Data are presented as mean ± SD (n = 3; ***, *p* < 0.001). **F** Mitochondrial fraction from MCF7 cells was incubated with 50 μg/mL of proteinase K in the absence or presence of 1% Triton X-100 for 30 min. Samples were subjected to western blotting analysis.

Our previous report has revealed that TRIP-Br1 protein is localized to the mitochondria of various cancer cell lines 27,28. The current study also showed that TRIP-Br1 was more predominantly found in the mitochondria of human breast cancer (MCF7 and MDA-MB-231) and colon cancer (HCT116) cell lines than in a normal breast cell line (MCF10A) (Figures 1A-1B). Similar results were obtained via immunofluorescence analysis, showing that endogenous mitochondrial TRIP-Br1 was co-localized with Mitotracker (Figure 1C). Next, mitochondrial TRIP-Br1 protein levels were further compared between MCF7 cancer and MCF10A normal cell lines. Although total levels of TRIP-Br1 protein were slightly higher in MCF7 cells, mitochondrial TRIP-Br1 levels were substantially higher in MCF7 cells than in MCF10A normal cells (Figures 1D-1E). MCF10A normal cells showed substantially higher levels of TRIP-Br1 in cytosol than in mitochondria. In a further study, sub-localization of TRIP-Br1 in mitochondrial compartment was analyzed by adding proteinase K to mitochondrial fractionized sample of MCF7. Proteinase K treatment degraded TRIP-Br1 and VDAC1, a mitochondrial outer membrane protein, but not cytochrome c, a mitochondrial inner membrane protein, suggesting that TRIP-Br1 was accumulated in mitochondrial outer membrane (Figure 1F).

Collectively, these data suggest the presence of a significant amount of TRIP-Br1 protein in mitochondria of cancer cells, mainly localized in the outer membrane.

### ROS-triggered TRIP-Br1 translocation into mitochondria and TRIP-Br1-mediated ROS suppression in response to anticancer drug treatment

**Figure 2:**
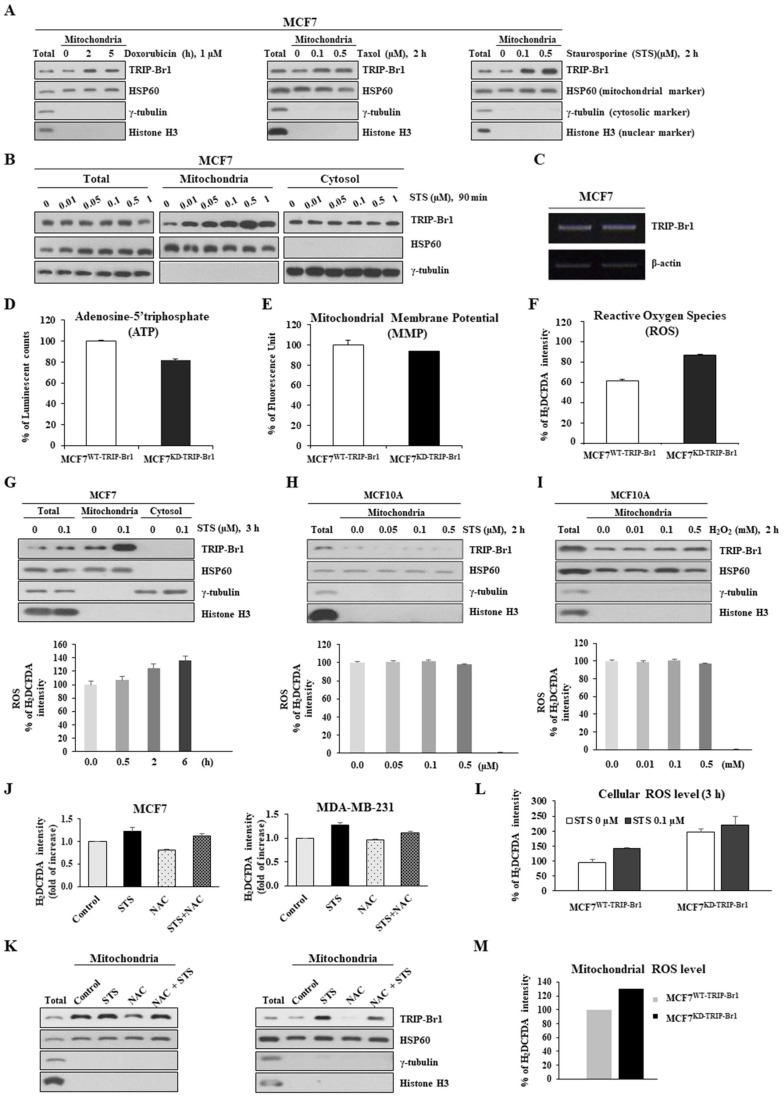
** Up-regulated TRIP-Br1 protein level in mitochondria in response to anticancer drug treatment and TRIP-Br1-mediated ROS suppression in cancer cells. A** MCF7 cells were treated with three different anticancer drugs for indicated time periods and concentrations, followed by mitochondrial fractionation. **B** MCF7 cells were treated with STS at different concentrations (0.01~1 μM) for 90 min followed by mitochondrial fractionation. **C** MCF7 cells were treated with 0.1 μM of STS for 4 h. Reverse transcription (RT)-PCR analysis was performed with TRIP-Br1-specific primer pairs (See Materials and Methods) using β-actin as an internal control. **D-F** MCF7 cells (1×10^6^) were cultured for 24 h and intracellular levels of ATP, MMP, and ROS were measured as indicated in Materials and Methods. Results are presented as mean ± SD (n = 5). **G-I** MCF7 and MCF10A cells were cultured for 24 h and then treated with STS or H_2_O_2_ at indicated concentrations or time periods. Resulting cells were used for mitochondrial fractionation and ROS measurement. **J-K** Cells were pre-incubated with or without NAC, an ROS scavenger, for 30 min (5 mM for MCF7 and 1 mM for MDA-MB-231) and then treated with 0.1 μM of STS for 45 min. Each fraction of sample lysate was subjected to western blotting analysis. ROS levels were measured with the same sample. **L-M** MCF7 cells were treated with 0.1 μM of STS for 3 h. Cellular ROS (**L**) or mitochondrial ROS (**M**) levels were then measured. Data are presented as mean ± SD (n = 6).

TRIP-Br1 protein levels in mitochondria after treatment with three different anticancer drugs were analyzed using the MCF7 cell line, a well-known cancer cell line that is highly resistant to many types of anticancer drugs 2. TRIP-Br1 protein levels were increased significantly in the mitochondria after treatment with all three anticancer drugs even only after a short-term exposure (2~5 h) (Figure 2A). Especially, staurosporine (STS) greatly increased TRIP-Br1 protein levels in the mitochondria, but not total cellular protein levels of TRIP-Br1 (Figures 2A-2B). In fact, the mRNA level of TRIP-Br1 was not changed after 4 h treatment with anticancer drugs (Figure 2C), suggesting mitochondrial translocation of existing TRIP-Br1 rather than *de novo* synthesis of TRIP-Br1 after a short exposure to STS.

Next, we investigated the trigger of TRIP-Br1 translocation into mitochondria and the role of TRIP-Br1 in the mitochondria of cancer cells upon treatment with anticancer drugs. To this end, we first determined the effect of TRIP-Br1 on three representative mitochondrial functions. TRIP-Br1 knock-downed MCF7 (MCF7^KD-TRIP-Br1^) cells showed slightly lower levels of ATP and MMP, but higher ROS levels than TRIP-Br1 wild-type MCF7 (MCF7^WT-TRIP-Br1^) cells (Figures 2D-2F). Anticancer treatment is known to induce substantially higher levels of ROS in cancer cells. However, some malignant cells can overcome this ROS stress. Thus, we initially focused on intracellular ROS contents. MCF7 cells were treated with 0.1 μg/mL of STS for 6 h. Intracellular ROS content was then evaluated. Interestingly, mitochondrial TRIP-Br1 expression was increased following the accumulation of ROS (Figure 2G). However, levels of both mitochondrial TRIP-Br1 expression and ROS in MCF10A normal cells did not change after STS treatment (Figure 2H). MCF10A cells did not respond to treatment with H_2_O_2_ (a major ROS contributor) either (Figure 2I). These data indicate that mitochondrial TRIP-Br1 protein levels are more sensitive to cellular ROS levels in cancer cells, implying that ROS might trigger mitochondrial translocation of TRIP-Br1 in cancer cells. This possibility was further tested using MCF7 and MDA-MB-231 cell lines treated with NAC, an ROS scavenger, as well as STS. Interestingly, a reduced mitochondrial TRIP-Br1 level was found in cells treated with both STS and NAC than in cells treated with STS alone (Figures 2J-2K). A further study showed substantially higher levels of cellular and mitochondrial ROS in TRIP-Br1 suppressed MCF7 cells under both basal conditions and upon STS exposure (Figures 2L-2M), suggesting that TRIP-Br1 could suppress cellular ROS levels in cancer cells.

Taken together, these findings suggest that STS treatment could elevate ROS production, which induces mitochondrial translocation of TRIP-Br1 to suppress ROS generation in cancer cells.

### Mitochondrial translocation of TRIP-Br1 via dephosphorylation by protein phosphatase 2A (PP2A)

**Figure 3:**
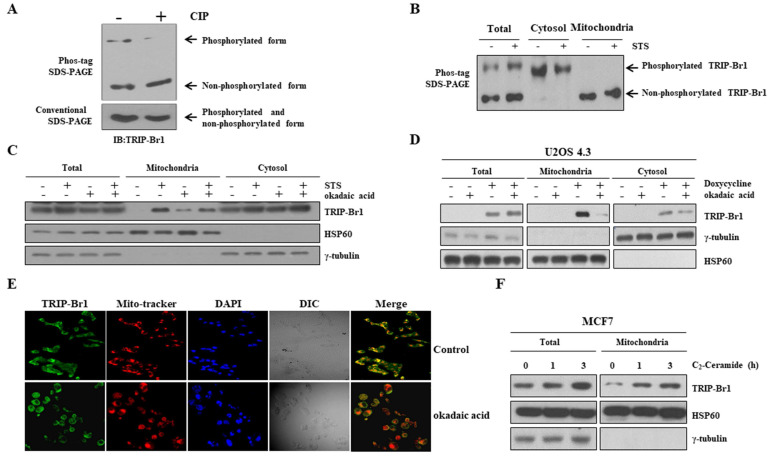
** Mitochondrial translocation of TRIP-Br1 via PP2A-mediated dephosphorylation upon STS treatment. A** Phosphorylation status of TRIP-Br1 was analyzed using a Phos-tag SDS-PAGE gel. Lysates of MCF7 cells were prepared and then exposed to CIP for 1 h at 37°C, followed by 8 % SDS-PAGE with 20 μM of phos-tag and immunoblotting analysis. Conventional SDS-PAGE was also performed with the same lysate as a control. **B** MCF7 cells were treated with 0.1 μM STS for 90 min followed by subcellular fractionation. Fractionated samples were subjected to Phos-tag SDS-PAGE. **C** MCF7 cells were treated with STS (0.1 μM) and/or okadaic acid (50 nM) for 3 h. Fractionated samples were subjected to western blot analysis. **D** U2OS 4.3 osteosarcoma cells were pre-incubated with or without doxycycline (1 μg/mL) to induce TRIP-Br1 expression for 24 h before treatment with okadaic acid (50 nM) for 24 h.** E** MCF7 cells were treated with okadaic acid (50 nM) for 16 h and incubated with Mitotracker (red) for 30 min. TRIP-Br1 (green) and Mitotracker (red) were visualized under a fluorescence microscope. **F** MCF7 cells were exposed to C_2_-Ceramide (10 μM) in a time-dependent manner followed by mitochondrial fractionation.

Next, we investigated the mechanism of TRIP-Br1 translocation into mitochondria. Using a protein localization prediction program (TargetP 1.1 Server), TRIP-Br1 showed a low possibility of independent mitochondrial localization because TRIP-Br1 did not contain a mitochondrial target sequence (MTS) (Supplementary Figure 1). Instead, we hypothesized that it might be mediated via post-translational modification (PTM) because many different types of PTM are strongly related to sub-cellular re-localization. Interestingly, our Phos-tag SDS-PAGE analysis revealed phosphorylated and dephosphorylated forms of TRIP-Br1 compared with conventional SDS-PAGE after STS treatment (Figure 3A). Further tests showed that the TRIP-Br1 phosphorylated form was present in the cytosol, whereas the dephosphorylated form existed in the mitochondria (Figure 3B). We also hypothesized that TRIP-Br1 might be dephosphorylated by PP2A. It has been proposed that PP2A can dephosphorylate TRIP-Br1 and stabilize its expression 41. In addition, PP2A can be activated via oxidative stress such as high ROS generation to participate in many signaling pathways of mammalian cells 42-47. In fact, our mitochondrial fractionation experiment showed that TRIP-Br1 translocation into mitochondria was blocked when cells were treated with okadaic acid, a PP2A specific inhibitor (Figure 3C) 48,49. Our hypothesis was further tested in U2OS 4.3 osteosarcoma cells, in which TRIP-Br1 expression could be induced by doxycycline 50. Treatment with doxycycline only induced higher levels of mitochondrial TRIP-Br1, whereas treatment with both doxycycline and okadaic acid remarkably decreased levels of mitochondrial TRIP-Br1 (Figure 3D). Immunofluorescence data also showed that TRIP-Br1 protein levels were decreased significantly in mitochondria after okadaic acid treatment (Figure 3E). In contrast, C_2_-ceramide, a potent activator of PP2A 51,52, enhanced mitochondrial translocation of TRIP-Br1 after treatment (Figure 3F).

Collectively, these data strongly suggest that translocation of TRIP-Br1 into mitochondria is induced by PP2A-mediated dephosphorylation upon anticancer treatment, at least partly.

### TRIP-Br1-mediated PCD suppression in cancer cells via induction of autophagy

It is known that cancer cells usually produce significant amounts of ROS following anticancer treatment, which usually trigger mitochondrial damage and enhance the toxicity to cancer cells. Our data also showed that STS treatment enhanced ROS levels, whereas TRIP-Br1 suppressed cellular ROS generation and finally PCD in cancer cells. Therefore, we hypothesized that TRIP-Br1 could repress cellular ROS generation by inducing mitophagy for rapid clearance of damaged mitochondria. Thus, we evaluated effects of TRIP-Br1 on autophagy and mitophagy.

**Figure 4:**
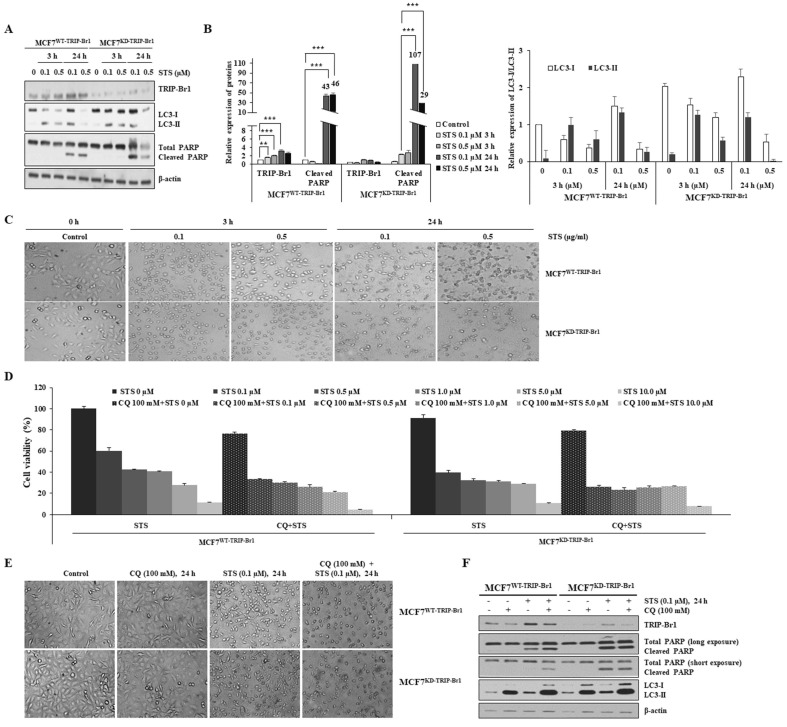
** Suppression of STS-mediated cell death by TRIP-Br1 via induction of autophagy**. **A-B** MCF7^WT-TRIP-Br1^ and MCF7^KD-TRIP-Br1^ cells were cultured for 24 h and then treated with 0.1 or 0.5 µM of STS for 3 h or 24 h. Western blot was performed and β-actin was used as a loading control. Data are presented as mean ± SD (n = 3; **, *p* < 0.01, ***, *p* < 0.001). **C** MCF7^WT-TRIP-Br1^ and MCF7^KD-TRIP-Br1^ cells were cultured in 100 mm culture dishes for 24 h and then treated with 0.1 or 0.5 µM of STS for 3 h or 24 h, respectively. Cell morphology was observed using an optical microscope with an IS capture software (KI-400F; Korea Lab Tech, Seongnam, South Korea) at ×100 magnification. The experiment was repeated three times. Images represent the average cell morphology. **D** MCF7^WT-TRIP-Br1^ and ^MCF7KD-TRIP-Br1^ cells were pretreated with CQ (100 mM) for 6 h, followed by STS treatment at indicated concentrations for 24 h. Cell viability was measured using a CellTiter-Glo 3D cell viability assay kit. Data are expressed as mean ± SD (n = 4). **E** Morphological changes in MCF7^WT-TRIP-Br1^and MCF7^KD-TRIP-Br1^ cells were observed after treatment with CQ (100 mM) for 24 h, followed by STS (0.1 μM) treatment for 24 h. **F** MCF7^WT-TRIP-Br1^ and MCF7^KD-TRIP-Br1^ cells were pre-treated with CQ (100 mM) for 3 h, followed by STS (0.1 μM) treatment for 24 h. Each protein level was detected via immunoblotting using corresponding antibody.

At first, effects of TRIP-Br1 on autophagy and PCD were analyzed using MCF7^TRIP-Br1-WT^ and MCF7^TRIP-Br1-KD^ cells following a short-term (3 h) and a long-term (24 h) exposure to STS (0.1 and 0.5 μg/mL). While short-term treatment with STS (3 h) induced translocation of TRIP-Br1 into mitochondria (Figure 2B), long-term exposure to STS (24 h) slightly increased TRIP-Br1 protein level (Figures 4A-4B). MCF7^WT-TRIP-Br1^ cells showed higher conversion ratio from LC3-I to LC3-II, but lower cleaved PARP level upon treatment with 0.1 µM STS for 24 h, implying that highly activated autophagy might repress apoptosis. Substantially lower levels of LC3 and PARP proteins were detected in MCF7^KD-TRIP-Br1^ cells treated with 0.5 µM STS due to a very high level of cell death (Figures 4A-4C).

To test whether TRIP-Br1 could suppress STS-induced PCD via induction of active autophagy, autophagy was blocked using chloroquine (CQ), a well-known inhibitor of autophagy 53. Substantially higher levels of MCF7^WT-TRIP-Br1^ cells were viable than MCF7^KD-TRIP-Br1^ cells following treatment with only STS (Figure 4D). However, cell viability was decreased dramatically for MCF7^WT-TRIP-Br1^ cells upon treatment with both STS and CQ. For MCF7^KD-TRIP-Br1^ cells, a similar lower level of cell viability was detected regardless of CQ treatment (Figure 4D). Morphological analysis also yielded the same results (Figure 4E). Western blot analysis revealed substantially lower levels of cleaved PARP in MCF7^WT-TRIP-Br1^ cells than in MCF7^KD-TRIP-Br1^ cells after STS treatment. However, levels of cleaved PARP were greatly increased by combined treatment of both STS and CQ (Figure 4F). Again, PARP cleavage in MCF7^KD-TRIP-Br1^ cells did not change drastically between before and after CQ treatment (Figure 4F).

These data strongly indicate that TRIP-Br1 can suppress PCD by activating autophagy.

### TRIP-Br1-mediated PCD suppression in cancer cells via induction of mitophagy

**Figure 5:**
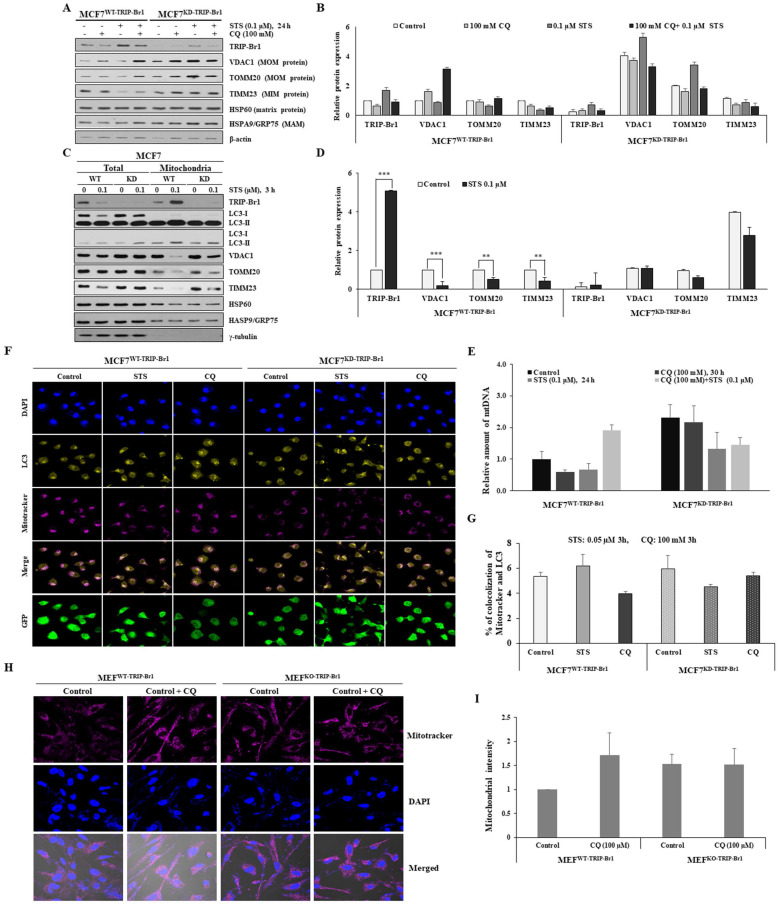
** Suppression of STS-mediated cell death by TRIP-Br1 via induction of mitophagy. A-B** MCF7^WT-TRIP-Br1^ and MCF7^KD-TRIP-Br1^ cells were pre-treated with CQ (100 mM), followed by STS (0.1 μM) for 24 h. Each protein level was detected via immunoblotting using the corresponding antibody. **C-D** MCF7^WT-TRIP-Br1^ and MCF7^KD-TRIP-Br1^ cells were cultured in the absence or presence of STS (0.1 μM) for 3 h. Resulting cells were used for mitochondrial fractionation and western blotting analysis as mentioned in Materials and Methods. Data are expressed as mean ± SD (n = 3; **, *p* < 0.01; ***, *p* < 0.001). **E** Relative quantification of mtDNA amount in MCF7^WT-TRIP-Br1^ and MCF7^KD-TRIP-Br1^ cells was performed using real-time (RT)-PCR. Cells were pre-treated with or without 100 mM CQ for 6 h and then treated with 0.1 µM STS for 24 h. Relative mtDNA levels were normalized to GAPDH and a graphical representation of the summary data is presented. Data are expressed as mean ± SD (n = 3). **F** MCF7^WT-TRIP-Br1^ and ^MCF7KD-TRIP-Br1^ cells were grown in confocal dishes for 24 h. These cells were incubated with STS (0.1 μM) or CQ (100 mM) for 6 h. They were then incubated with 1 µM of Mitotracker dye for 30 min. Cells were stained with LC3 (yellow) and Mitotracker (red). Representative fluorescent images were visualized with a confocal microscope (scale bars, 20 μM). The co-localization of mitochondria and LC3 was measured by counting more than 100 cells in ImageJ per experiment for each group. Data represent the mean value of three independent experiments. **H-I** TRIP-Br1 wild-type MEF (MEF^WT-TRIP-Br1^) and TRIP-Br1-null MEF (MEF^KO-TRIP-Br1^) were cultured in confocal dishes for 24 h, followed by staining with Mitotracker (100 nM) for 30 min. Representative fluorescent images were visualized under an immunofluorescence microscope (scale bars, 20 μm). Mitochondria intensity with red staining was quantified using ImageJ.

Next, the effect of TRIP-Br1 on mitophagy progression was evaluated using early and advanced mitophagy markers to indicate the degradation of mitochondrial proteins residing in outer (VDAC1 and TOMM20) and inner (TIMM23) membranes, respectively. It has been proposed that mitochondrial outer membrane (MOM) proteins are degraded by proteasome prior to the induction of mitophagy. These proteins are known as early mitophagy markers 54. Our data showed that two early mitophagy markers (VDACI and TOMM20) and one advanced mitophagy maker (TIMM23) were substantially lower in MCF7^WT-TRIP-Br1^ cells than in MCF7^KD-TRIP-Br1^ cells upon STS treatment (Figures 5A-5B). However, no major difference existed between MCF7^WT-TRIP-Br1^ and MCF7^KD-TRIP-Br1^ cells after combined treatment with STS and CQ (Figures 5A-5B). Levels of mitochondrial membrane proteins were significantly higher in MCF7^KD-TRIP-Br1^ cells regardless of treatment with CQ (Figures 5A-5B). Expression levels of heat shock protein 60 (HSP60, a mitochondrial matrix protein) and heat shock protein family A (HSP70) member 9 (HSPA9/GRP75, a mitochondrial associated membrane (MAM) protein) as control proteins were not altered by STS treatment. Their levels in MCF7^WT-TRIP-Br1^ and MCF7^KD-TRIP-Br1^ cells showed no substantial differences either (Figure 5A). These data suggest the delayed degradation of mitochondrial membrane proteins and therefore a significant impairment of mitochondrial clearance in MCF7^KD-TRIP-Br1^ cells upon STS treatment.

Mitochondrial fractionation data also showed that protein levels of three mitochondrial membrane proteins (VDAC1, TOMM20, and TIMM23) were substantially lower in the mitochondria of MCF7^WT-TRIP-Br1^ cells than in the mitochondria of MCF7^KD-TRIP-Br1^ cells for both control and test samples subjected to a short-term treatment with 0.1 µM STS (Figures 5C-5D). Expression levels of these three mitochondrial proteins were greatly decreased in the mitochondria of MCF7^WT-TRIP-Br1^ cells, but only slightly decreased in the mitochondria of MCF7^KD-TRIP-Br1^ cells following a short-term exposure to 0.1 µM STS (Figures 5C-5D). We consistently observed significantly higher levels of these proteins in MCF7^KD-TRIP-Br1^ cells than in MCF7^WT-TRIP-Br1^ cells in the absence or presence of STS. Again, HSP60 and HSPA9/GRP75 protein levels in MCF7^WT-TRIP-Br1^ and MCF7^KD-TRIP-Br1^ cells showed no significant differences (Figure 5C). These findings suggest that TRIP-Br1 can strongly activate mitophagy upon STS treatment.

These findings were further reinforced by monitoring mitochondrial mass. The relative quantification of mtDNA levels upon STS treatment was achieved using real-time (RT)-PCR (Figure 5E). A markedly reduced mtDNA level was found in MCF7^WT-TRIP-Br1^ cells than in MCF7^KD-TRIP-Br1^ cells upon STS treatment as well as in basal conditions. However, mtDNA level was increased by combined treatment with CQ and STS (Figure 5E). It was found that mtDNA level was also decreased in MCF7^KD-TRIP-Br1^ cells after STS treatment, showing no significant difference between before and after CQ treatment (Figure 5E). It has been suggested that reduced mtDNA level can attenuate the respiratory chain, resulting in decreased ROS production 55. This suggestion is consistent with our data showing higher ROS levels in TRIP-Br1 suppressed MCF7 cells. Co-immunofluorescence analysis also revealed a higher co-localization of mitochondria and LC3 in MCF7^WT-TRIP-Br1^ cells than in MCF7^KD-TRIP-Br1^ cells upon STS treatment, suggesting higher levels of mitophagy in wild-type TRIP-Br1 expressing MCF7 cells (Figures 5F-5G).

Our *in vitro* data showed fewer mitochondria in MCF7^WT-TRIP-Br1^ cells than in MCF7^WT-TRIP-Br1^ cells even in the control group probably due to a higher basal level of mitophagy in MCF7^WT-TRIP-Br1^ cells (Supplementary Figure 2). Consistent with our *in vitro* data, a similar result was obtained with MEF cells isolated from TRIP-Br1 wild-type (MEF^WT-TRIP-Br1^) and knock-out mice (MEF^KO-TRIP-Br1^). Lower levels of mitochondria were detected in MEF^WT-TRIP-Br1^ cells than in MEF^KD-TRIP-Br1^ cells under a fluorescence microscope (Figures 5H-5I). However, CQ treatment induced the accumulation of mitochondria in MEF^WT-TRIP-Br1^. Again, no difference was detected in MEF^KD-TRIP-Br1^ cells regardless of CQ. These data suggest higher levels of mitophagy in MEF^WT-TRIP-Br1^ than in MEF^KD-TRIP-Br1^ cells. TRIP-Br1 knock-out was confirmed by PCR (Supplementary Figure 3).

Collectively, these findings suggest that TRIP-Br1 can strongly activate mitophagy upon STS treatment.

### Enhanced TRIP-Br1-mediated mitophagy via up-regulation of lysosomal proteins, cathepsins B and D

**Figure 6:**
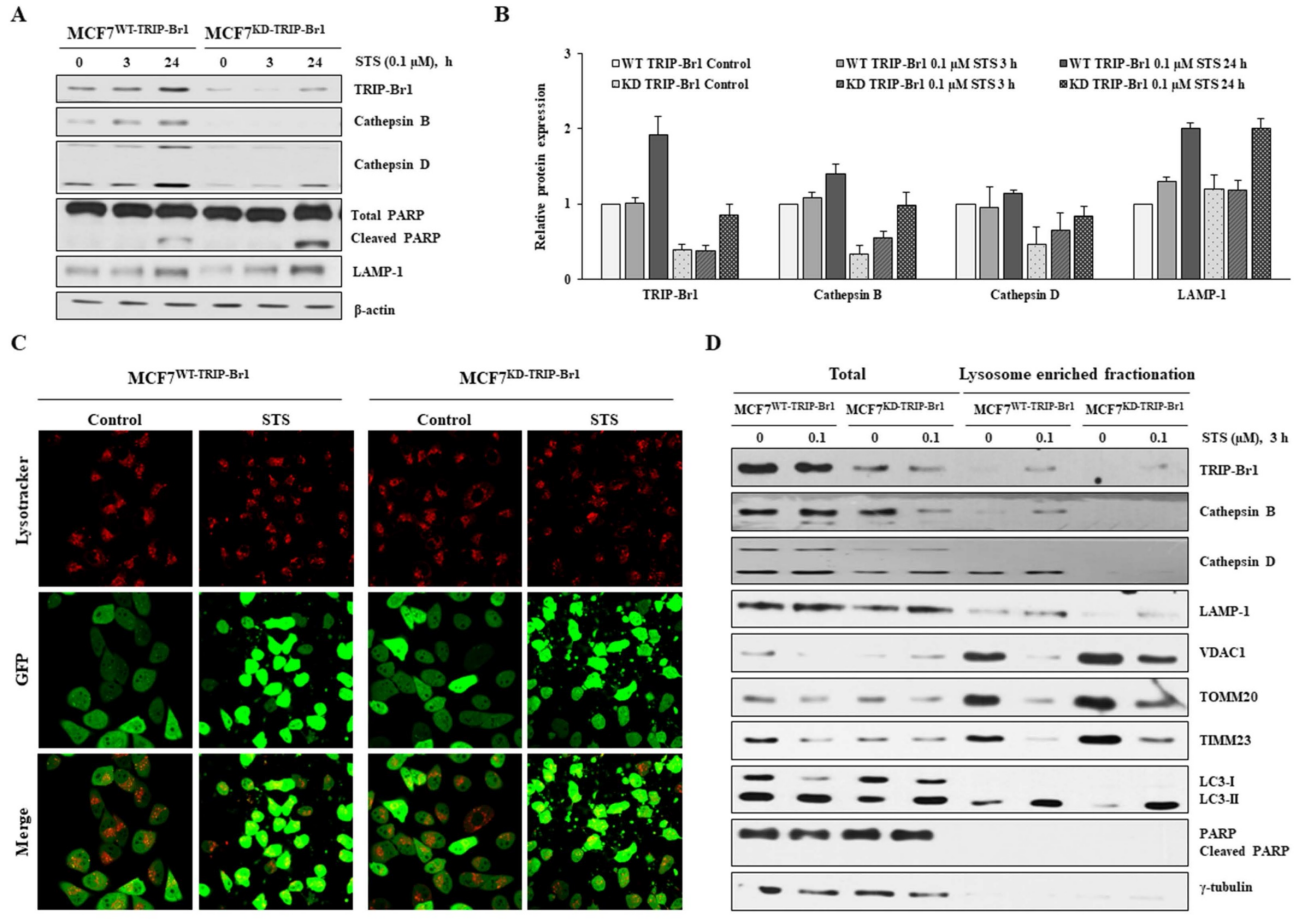
** Enhanced TRIP-Br1-mediated mitophagy via up-regulation of lysosomal proteases cathepsins B and D. A-B** MCF7^WT-TRIP-Br1^ and MCF7^KD-TRIP-Br1^ cells were treated with 0.1 µM STS for 3 h and 24 h. The precursor and mature forms of cathepsins B and D were determined via immunoblotting analysis. β-actin was used as an internal control. Data are presented as mean ± SD (n = 3). **C** MCF7^WT-TRIP-Br1^ and MCF7^KD-TRIP-Br1^ cells were cultured in confocal dishes for 24 h and then incubated with Lysotracker (Red) for 90 min. These cells were captured using a confocal microscope. Quantification of red fluorescence intensity of Lysotracker is shown. Values are expressed as mean ± SD of three independent experiments with each count of no less than 100 cells. **D** Both cell lines were cultured for 24 h and then treated with 0.1 μM STS for 3 h. Cells were harvested and lysosomal fractions were isolated as described in Materials and Methods. **γ**-Tubulin was used as a cytosolic marker.

Lysosome is a vital cellular organelle in the mitophagy process 56. Lysosome contains 11 cathepsins, the most abundant and essential lysosomal proteases. Of all lysosomal proteases, the most abundant cathepsins B and D are highly overexpressed in breast cancer cells to regulate breast cancer cell growth and metastasis 57,58. Therefore, we investigated the effect of TRIP-Br1 on lysosomal function in terms of cathepsins. Markedly higher levels of cathepsins B and D proteins were detected even in the control (Figures 6A-6B). Their levels were increased substantially, whereas lower levels of cleaved PARP were found in TRIP-Br1 wild-type cells than in TRIP-Br1 knock-downed cells after 24 h of STS treatment (Figures 6A-6B). However, lysosomal membrane protein LAMP-1 levels were increased to a similar extent in both cell lines (MCF7^WT-TRIP-Br1^ and MCF7^KD-TRIP-Br1^ cells) after STS treatment (Figures 6A-6B). In addition, no significant difference was found in the intensity of lysotracker between MCF7^WT-TRIP-Br1^ and MCF7^KD-TRIP-Br1^ cells, implying not much difference in the number or the size of lysosomes between the two cell lines (Figure 6C).

Lysosome enriched fractionation experiment also revealed significantly higher levels of cathepsins B and D in both total and lysosomal fractionations of MCF7^WT-TRIP-Br1^ cells compared with those of MCF7^KD-TRIP-Br1^ cells (Figure 6D). Notably, the adopted lysosome enriched fraction contained autophagolysosome as well as pure lysosome. The crude lysosomal fraction still contained portions of mitochondria, suggesting basal level of mitophagy. Therefore, mitochondrial proteins (VDAC1, TOMM20, and TIMM23) were also found in lysosome-enriched fractions of both MCF7^WT-TRIP-Br1^ and MCF7^KD-TRIP-Br1^ cells based on the detection of only LC3-II, but not LC3-I in lysosome-enriched fractions in control groups as well as in STS-treated groups. An inverse relationship was found between mitochondrial marker proteins and the expression of cathepsins B or D (Figure 6D).

Our findings demonstrate that TRIP-Br1 appears to enhance mitophagy at least via up-regulation of cathepsins B and D under both control and STS-treated conditions.

## Discussion

Targeting cancer cell-specific autophagy/mitophagy may facilitate the development of novel strategies for the treatment of various types of cancer. Our results may provide one clue to target autophagy/mitophagy mechanisms in cancer research. We found that up-regulated mitochondrial TRIP-Br1 inhibited STS-mediated PCD of breast cancer cells by inducing mitophagy, at least partly.

A further study is needed to analyze the function of TRIP-Br1 in lysosomes, one of key players in mitophagy. Lysosome not only suppresses tumorigenesis in normal cells, but also shows oncogenic characteristics in cancer cells. Lysosome can induce anticancer drug resistance by blocking chemotherapeutic drugs from reaching their cellular targets 59. Cathepsins play a vital role in the degradation of intracellular proteins and organelles. In cancer cells, overexpression of cathepsins can promote cell migration, proliferation, invasion, and metastasis 56,57,60. Many studies have shown that lysosomal membrane permeability (LMP) can induce PCD and that LMP is strongly correlated with cancer. PCD stimulating stress factors such as ROS and anticancer drugs can induce LMP, which then triggers the release of cathepsins from the lysosomal lumen into the cytosol, thereby activating caspases. Cathepsins B and D can cleave Bid and induce its proteolytic activation, followed by induction of MOMP, resulting in cytochrome *c* release and apoptosome-dependent caspase activation. Thus, TRIP-Br1 may suppress LMP and PCD eventually by blocking lysosomal release of cathepsins B and D into the cytoplasm. This hypothesis needs to be further tested.

Degradation of outer or inner mitochondrial membrane can trigger mitophagy. We and others have shown that TRIP-Br1 is responsible for the ubiquitination and degradation of membrane proteins in conjunction with E3 ligases (XIAP, NEDD4-1) 28,34. Therefore, it would be interesting to test whether mitochondrial relocalization of TRIP-Br1 upon anticancer treatment can induce ubiquitination and degradation of MOM and MIM (e.g., VDAC1, TOMM20, TIMM23) in combination with unknown E3 ligases. In fact, we found that TRIP-Br1 positively regulated the expression of PARKIN, a vital E3 ligase in mitophagy. However, direct interaction between TRIP-Br1 and PARKIN was not detected (data not shown).

Defective mitochondria can be eliminated via mitoptosis and mitophagy, especially when mitophagy is inhibited. TRIP-Br1 might also be involved in mitoptosis. Mitoptosis involves both inner and outer mitochondrial membranes. During “inner mitochondrial membrane mitoptosis”, only the internal matrix and cristae are degraded, while the outer mitochondrial membrane remains intact 61. Our data revealed that a substantially lower level of TIMM23 was detected in MCF7^WT-TRIP-Br1^ cells after STS treatment, implying a possible induction of inner membrane mitoptosis.

In a further study, we tried to find out the phosphorylation site in TRIP-Br1 by making single point mutations of TRIP-Br1 protein. We and Zang *et al.* have predicted potential phosphorylaton sites (7 serine and 1 tyrosine) using bionformatic tools 41. Our data showed that all mutation did not induce an increase of TRIP-Br1 in mitochondria upon STS treatment (Supplementary Figure 4). However, we could not exclude the possibility of three-dimentinal distortion of TRIP-Br1 due to single amino acid change, which might affect TRIP-Br1 translocation. Thus, the exact phosphorylation site(s) responsible for TRIP-Br1 tranlocation needs to be discovered in the future.

Our current studies imply that TRIP-Br1 oncoprotein represents a possible candidate targeting cancer-specific mitophagy when combined with anticancer drugs to efficiently kill cancer cells.

## Materials and Methods

### Cell lines, cell culture, and cell treatment

All cancer cell lines were cultured in Dulbecco's modified Eagle's medium (DMEM; WelGENE, Korea) supplemented with 10% fetal bovine serum (FBS) (Gibco BRL) and 1% antibiotic-antimycotic solution (Gibco BRL, Cat#15240-062). A normal human MCF10A mammary epithelial cell line was grown as described elsewhere 27. All cells were maintained at 37°C in a humidified atmosphere with 95% air and 5% CO_2_. MCF7 and MDA-MB-231 human breast cancer cell lines were purchased from the American Type Culture Collection (ATCC). MCF7 cell lines with wild-type or knock-down TRIP-Br1 were established in our previous study 27. U2OS 4.3, a TRIP-Br1-inducible osteosarcoma cell line, was a kind gift from Dr. Rikiro Fukunaga (Kyoto University, Japan). Other reagents used in this study included staurosporine (STS) (A.G. Scientific, S-1016), chloroquine (CQ)(Sigma-Aldrich, C6628), okadaic acid (A.G. Scientific, O-1028), and C_2_-ceramide (Enzo Life science, BML-SL100).

### Cell viability analysis

Cell viability was analyzed using a CellTiter-Glo® 3D Cell Viability Assay (Promega, G9681), according to the manufacturer's instructions. Briefly, cells (2 × 10^4^ cells/well) were cultured in 96-well plates and then exposed to STS at indicated concentrations and time periods. These cells were vigorously mixed with a CellTiter-Glo 3D reagent for 5 min and incubated at room temperature for 30 min to stabilize luminescence. Cell viability was assessed by measuring the absorbance at luminescence in a GloMax® Discover (Promega). The analysis was conducted in triplicate.

### Western blot analysis

Western blot was performed as described in our previous study 27. Antibodies employed in this study were: anti-TRIP-Br1 (Enzo Life Sciences, ALX-804-645), anti-PARP (Cell Signaling Technology, #9542S), anti-cytochrome c (Santa Cruz Biotechnology, sc-7159), anti-CypA (Enzo Life Sciences, BML-SA296), anti-LC3 (Cell Signaling Technology, #2775S), anti-VDAC1 (Cell Signaling Technology, #4661), anti-TOMM20 (BD biosciences, 612278), anti-TIMM23 (BD biosciences, 611222), anti-HSP60 (Santa Cruz Biotechnology, sc-139661), anti-HSP70/GRP75 (Santa Cruz Biotechnology, sc-13967), anti-cathepsin B (Bioworld, BS3536), anti-cathepsin D (Bioworld, BS90201), anti-LAMP1 (Santa Cruz Biotechnology, sc-20011), anti-rabbit (Cell Signaling Technology, #7074S), and anti-mouse (Santa Cruz Biotechnology, sc-516102). Antibodies against γ-tubulin (Santa Cruz Biotechnology, sc-7396) and β-actin (Santa Cruz Biotechnology, sc-47778) were used to measure levels of γ-tubulin and β-actin as loading controls. Results of western blot analysis were semi-quantified using ImageJ software (ver. 1.51u; National Institutes of Health, USA). The relative intensity was compared to γ-tubulin or β-actin level and presented as bar graphs.

### Mitophagy assessment

Mitophagy progression was calculated by monitoring the disappearance of mitochondrial membrane proteins (VDAC1, TOMM20 and TIMM23) and counting mitochondrial DNA (mtDNA) content via real-time PCR 62. The degradation of VDAC1 and TOMM20 was used to monitor early mitophagy, whereas TIMM23 expression was used to determine advanced mitophagy 62.

### Real-time polymerase chain reaction (RT-PCR) for mtDNA determination

Mitochondrial DNA was quantified by extracting total DNA using a HiYield Plus^TM^ Genomic DNA Mini Kit (Real Biotech Corporation, #QBT100). Rsulting mtDNAs were quantified using a 2×SYBR Green PCR master mix (ThermoFisher, #4367659) on a StepOnePlus™ (Applied Biosystems) with the following primers: mtDNA-(FW) 5'-AGGACAAGAGAAATAAGGCC-3'/(REV)5'-TAAGAAGAGGAATTGAACCTCTGACTGTAA-3'. GAPDH-(FW) 5'-CTGGGCTACACTGAGCACCAG-3'/(REV) 5'-CCAGCGTCAAAGGTGGAG-3'.

### Reverse transcription-polymerase chain reaction (RT-PCR)

Total RNA was extracted with an RNeasy Minikit (Qiagen, Cat. 74106, Germany) following the manufacturer's instructions. For reverse transcription, 1 μg RNA of each sample was subjected to cDNA synthesis using an ImProm-II™ Reverse Transcription System (Promega, #A3800). PCR amplification was performed using an AccuPower PCR PreMix system (Bioneer, Korea). Each gene product was amplified using the following primer pairs: TRIP-Br1 cDNA-(FW) 5'-AGGACCTCAGCCACATTGAG-3' and (REV) 5'-GGTGCCCAAAGTTCATTGTC-3'. ACTB cDNA (FW) 5'-CCATCGAGCACGGCATCGTCACCA-3'/(REV) 5'-CTCGGTGAGGATCTTCATGAGGTAGT-3'.

### Measurement of cellular ATP

Cellular ATP levels were measured using a luminescent ATP detection assay kit (Abcam, ab113849) according to the manufacturer's protocol. ATP levels were measured by detecting luminescence using a GloMax® Discover multimode microplate reader (Promega Corporation, Madison, WI, USA).

### Measurement of mitochondrial membrane potential (MMP)

MMP was determined using a Mito-ID Membrane Potential Cytotoxicity kit (cat. No. ENZ-51019; Enzo Life Sciences) according to the manufacturer's instruction. The MMP was measured based on the resulting fluorescence with a Gemini XPA microplate reader at an excitation wavelength of 480 nm and an emission wavelength of 590 nm.

### Measurement of reactive oxygen species (ROS)

ROS were measured with a Gemini XPA microplate reader as previously described 27.

### Mitochondrial fractionation

Cells were collected into 15 mL conical tubes after centrifugation at 1000 rpm for 5 min, washed with ice-cold PBS, resuspended with hypotonic lysis buffer (220 mM mannitol, 10 mM HEPES, 2.5 mM PO_4_H_2_ K, 1 mM EDTA, 68 mM sucrose, and 1 mM PMSF), stored on ice for 10 min, and then centrifuged at 1000 rpm for 5 min at 4°C. Cell pellets were resuspended in the hypotonic lysis buffer and gently pipetted approximately 10-20 times every 15 min during 1 h incubation on ice, followed by centrifugation at 1500-2000 rpm for 5 min at 4°C to remove cellular debris. The supernatant was transferred to a fresh tube and centrifuged at 14,000 rpm for 5 min at 4°C. The supernatant and pellet represent cytosolic and mitochondrial fractions, respectively. Pellets were resuspended with RIPA buffer and used in western blot analysis.

### Preparation of lysosomal fractionations

Lysosomes were isolated emplyoing a lysosome isolation kit (Abcam, #ab234047) following the manufacturer's protocol. Briefly, cells were collected into 15 mL conical tubes and isolated using an isolation buffer. The supernatant was centrifuged at 500 ×g for 10 min at 4°C and layered onto a discontinuous density gradient. Lysosomes were further isolated via ultracentrifugation (Beckman, Optima XE-100) at 145,000 g for 2 h at 4°C. Lysosomal fractions were extracted from cell homogenates using a lysosome extraction kit (Sigma-Aldrich; LYSISO1). Cell homogenates were centrifuged at 1000 × g for 10 min at 4°C. The supernatant fraction was centrifuged at 20,000 × g for 20 min at 4°C to pellet lysosomes and other organelles. The supernatant was collected as the cytosolic fraction. Pellet fractions were subjected to additional centrifugation. The ultimate pellet, lysosomal fraction, was lysed using the lysis buffer described within the procedure. Samples were then subjected to western blot analysis.

### Phos-tag SDS-PAGE

Phosphorylated and non-phosphorylated TRIP-Br1 were analyzed using Phos-tag™ SDS-PAGE gel as described by the manufacturer. Briefly, total cells or mitochondrial samples were prepared with a lysis buffer (20 mM HEPES, 120 mM NaCl, 1% Triton X-100) containing protease inhibitor cocktail. Cell lysates were subjected to 8% SDS-PAGE containing 50 mM Phos-tag acrylamide (Wako Pure Chemical Industries, 30493521) and 20 μM of phos-tag, followed by immunoblotting analysis.

### Immunofluorescence and confocal imaging

Cells (5 × 10^4^ cells) were grown on a sterilized confocal dish (Coverglass-Bottom Dish, SPL. Cat#100350) for 24 h. Cells were incubated with Mitotracker (Invitrogen, #M22426) for 30 min and fixed with 4% formaldehyde for 30 min. These cells were then washed with PBS twice and incubated with TRIP-Br1 (Enzo Life Sciences, ALX-804-645) or LC3 (Cell Signaling Technology, #2775S) antibodies overnight at 4°C. These primary antibodies were detected with an anti-mouse IgG H&L (Alexa Fluor® 568) (Abcam, #ab175473) or an anti-rabbit IgG H&L (Alexa Fluor® 488) (Abcam, #ab150077). Nuclei were stained with DAPI (Invitrogen, Cat#P36931) for 10 min after washing with PBS. Colocalization between fluorophores was analyzed using ImageJ software (ver. 1.51u; National Institutes of Health, USA). For lysosomal confocal imaging, cells were incubated with 100 nM Lysotracker (Invitrogen, Cat# L12492) in a phenol-free medium for 90 min. Confocal images were obtained using a Zeiss confocal microscope (Nikon A1 confocal). Image manipulation and merging were performed using appropriate tools of the ImageJ software.

### Animal experiment

TRIP-Br1 knockout mice (RRID: MGI:4437096) in a C57BL/6 genetic background were provided by Dr. Huang (Hong Kong University of Science and Technology, Hong Kong, China). Mice strains were genotyped via PCR as described previously 63. To isolate mouse embryonic fibroblasts (MEFs), embryos were dissected and decapitated from 13.5-day pregnant mice expressing wild-type or knock-out TRIP-Br1. These tissues were washed with cold PBS, cut into pieces, and incubated with trypsin/EDTA. Cells were transferred to DMEM media supplemented with 10 % FBS after each incubation. These cells were maintained in DMEM media after non-adherent cells were removed.

### Statistical analysis

Data are presented as mean ± standard deviation (SD) from a minimum of three independent experiments. All statistical analyses were performed using Student's t-test to check two different groups. One-way ANOVA, followed by Bonferroni's multiple comparison was accustomed to compare multiple groups. SPSS statistics version 23 (IBM Corporation, Armonk, NY, USA) was used for all statistical analyses. *P* < 0.05 indicated statistically significant difference.

## Supplementary Material

Supplementary figures.

## Abbreviations

TRIP-Br1: Transcriptional regulator interacting with the PHD-bromodomain 1
PCD: programmed cell death
mtDNA: mitochondrial DNA
STS: staurosporine
CQ: Chloroquine
ROS: Reactive Oxygen Species
MOM: mitochondrial outer membrane
MIM: mitochondrial inner membrane
PP2A: protein phosphatase 2A
VDAC1: voltage dependent anion channel 1
TOMM20: translocase of outer mitochondrial membrane 20
TIMM23: translocase of inner mitochondrial membrane 23
LC3: Microtubule-associated protein 1A/1B-light chain 3
