# Supplementary material for: Inhibitory role of TRIP-Br1 oncoprotein in anticancer drug-mediated programmed cell death via mitophagy activation
Source: Int J Biol Sci. 2022 May 29;18(9):3859–73. doi: 10.7150/ijbs.72138 (PMC9254482; doi:10.7150/ijbs.72138)

Supplementary Figure 1. Prediction of TRIP-Br1 mitochondrial localization. MitoProt software program, a computational method to predict mitochondrial localization of target protein, was used to predict the possibility of TRIP-Br1 mitochondrial localization using the full-length TRIP-Br1 amino acid sequence. The Mitoprot software is available at ftp://ftp.biologie.ens.fr/pub/molbio. Based on the protein localization prediction program (TargetP 1.1 Server), VDCA1, a well-known mitochondrial protein with the number of amino acid residues similar to TRIP-Br1, was used as a control. VDAC1 has a high probability of export into mitochondria. However, TRIP-Br1 does not carry the typical mitochondrial leader sequence. Therefore, it has a low possibility of mitochondrial translocation.

Supplementary Figure 2. A,B MCF7$^{WT\text{-}TRIP\text{-}Br1}$ and MCF7$^{KD\text{-}TRIP\text{-}Br1}$ cells were cultured in a confocal dish for 24 h, followed by staining with Mitotracker dye (100 nM) for 30 min. TRIP-Br1 localization in mitochondria was determined via two-color immunofluorescence microscopy using anti-TRIP-Br1 (yellow) and Mitotracker dyes (red). These cells were visualized under a confocal microscope. Representative fluorescent images are visualized with confocal microscopy (scale bar, 20 μm).

Supplementary Figure 3. Mice strains were genotyped via PCR as described previously [63].

Supplementary Figure 4. Potential phosphorylation sites amino acid sequences of TRIP-Br1 protein predicted with NetPhos 2.0 Server. Targeted potential phosphorylation sites were mutated by changing serine to alanine. Mitochondrial TRIP-Br1 levels are shown after STS treatment.

Supplementary Figure 1

TRIP-Br1

Input sequence length : 233 aa

VALUES OF COMPUTED PARAMETERS

Net charge of query sequence	: -28
Analysed region	: 10
Number of basic residues in targeting sequence	: 5
Number of acidic residues in targeting sequence	: 0
**Cleavage site**	**: not predictable**
Cleaved sequence	:

HYDROPHOBIC SCALE USED

		GES	KD	GVH1	ECS
H17	:	1.176	1.353	0.185	0.551
MesoH	:	-0.031	0.468	-0.183	0.295
MuHd_075	:	16.929	13.174	4.465	4.026
MuHd_095	:	16.679	11.844	5.013	4.232
MuHd_100	:	14.339	11.578	4.121	3.290
MuHd_105	:	18.576	12.091	5.196	3.839
Hmax_075	:	-25.083	-7.233	-7.833	-2.053
Hmax_095	:	-21.000	-4.987	-7.951	-1.015
Hmax_100	:	-26.800	-7.600	-8.948	-1.800
Hmax_105	:	-22.400	-4.783	-8.199	-1.237

Probability of export to mitochondria: 0.0608

Supplementary Figure 2

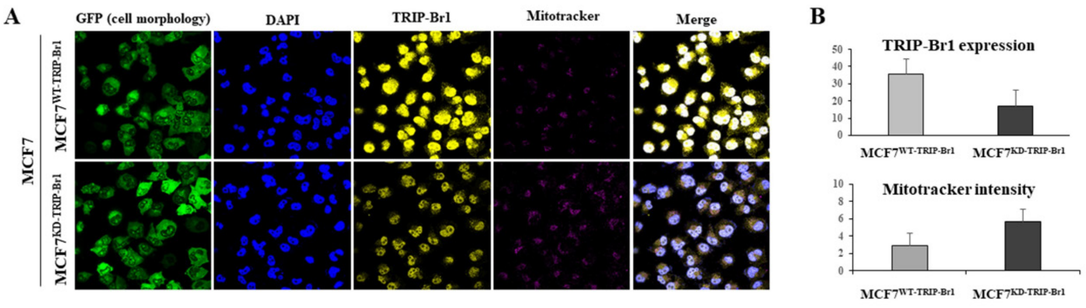

Supplementary Figure 3

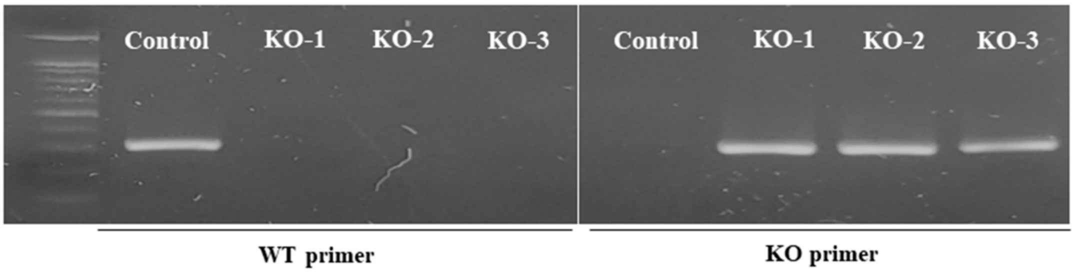

Supplementary Figure 4

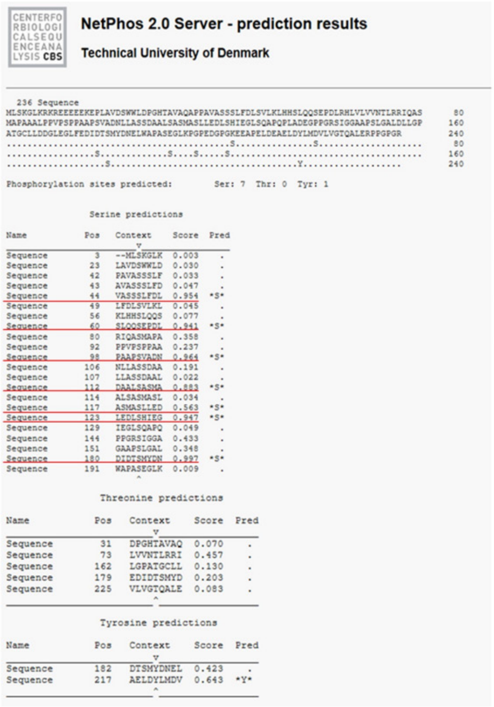

NetPhos 2.0 Server - prediction results

Technical University of Denmark

```
  236 Sequence
MLSKGLKRKREEEEEKEPLAVDSWWLDPGHTAVAQAPPAVASSSLFDLSVLKLHHSLQQSEPDLRHLVLVVNTLRRIQAS     80
MAPAAALPPVPSPPAAPSVADNLLASSDAALSASMASLLEDLSHIEGLSQAPQPLADEGPPGRSIGGAAPSLGALDLLGP    160
ATGCLLDDGLEGLFEDIDTSMYDNELWAPASEGLKPGPEDGPGKEEAPELDEAELDYLMDVLVGTQALERPPGPGR        240
```

Phosphorylation sites predicted: Ser: 7 Thr: 0 Tyr: 1

Serine predictions

Name	Pos	Context	Score	Pred
Sequence	3	--MLSKGLK	0.003	.
Sequence	23	LAVDSWWLD	0.030	.
Sequence	42	PAVASSSLF	0.033	.
Sequence	43	AVASSSLFD	0.047	.
Sequence	44	VASSSLFDL	0.954	*S*
Sequence	49	LFDLSVLKL	0.045	.
Sequence	56	KLHHSLQQS	0.077	.
Sequence	60	SLQQSEPDL	0.941	*S*
Sequence	80	RIQASMAPA	0.358	.
Sequence	92	PPVPSPPAA	0.237	.
Sequence	98	PAAPSVADN	0.964	*S*
Sequence	106	NLLASSDAA	0.191	.
Sequence	107	LLASSDAAL	0.022	.
Sequence	112	DAALSASMA	0.883	*S*
Sequence	114	ALSASMASL	0.034	.
Sequence	117	ASMASLLED	0.563	*S*
Sequence	123	LEDLSHIEG	0.947	*S*
Sequence	129	IEGLSQAPQ	0.049	.
Sequence	144	PPGRSIGGA	0.433	.
Sequence	151	GAAPSLGAL	0.348	.
Sequence	180	DIDTSMYDN	0.997	*S*
Sequence	191	WAPASEGLK	0.009	.

Threonine predictions

Name	Pos	Context	Score	Pred
Sequence	31	DPGHTAVAQ	0.070	.
Sequence	73	LVVNTLRRI	0.457	.
Sequence	162	LGPATGCLL	0.130	.
Sequence	179	EDIDTSMYD	0.203	.
Sequence	225	VLVGTQALE	0.083	.

Tyrosine predictions

Name	Pos	Context	Score	Pred
Sequence	182	DTSMYDNEL	0.423	.
Sequence	217	AELDYLMDV	0.643	*Y*

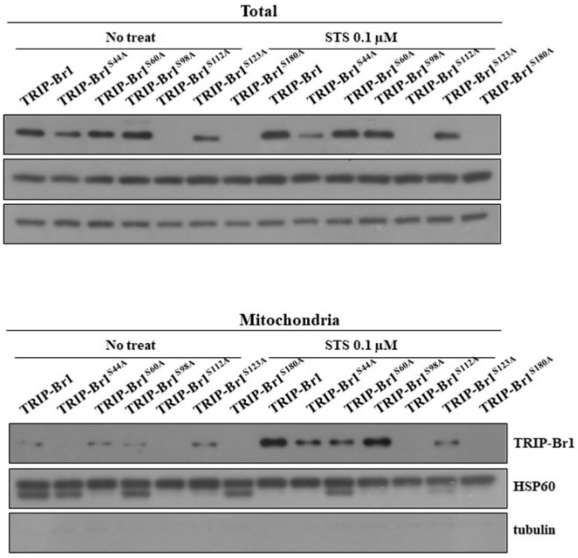

Supplement: Supplementary file 1 — Supplementary figures. [file ijbsv18p3859s1.pdf]
